# Serum neurofilament light chain as outcome marker for intensive care unit patients

**DOI:** 10.1007/s00415-020-10277-9

**Published:** 2020-10-23

**Authors:** Anna Lena Fisse, Kalliopi Pitarokoili, David Leppert, Jeremias Motte, Xiomara Pedreiturria, Ludwig Kappos, Ralf Gold, Jens Kuhle, Min-Suk Yoon

**Affiliations:** 1 Department of Neurology, St. Josef-Hospital, Ruhr-University Bochum, Gudrunstrasse 56, 44791 Bochum, Germany; 2 Departments of Medicine and Clinical Research, Neurologic Clinic and Policlinic, University Hospital and University of Basel, Basel, Switzerland; 3 Department of Neurology, Evangelisches Krankenhaus Hattingen, Hattingen, Germany

**Keywords:** Neurofilament, Intensive care unit, Outcome, Critical illness polyneuromyopathy

## Abstract

**Objective:**

Neurofilament light chain (NfL) in serum indicates neuro-axonal damage in diseases of the central and peripheral nervous system. Reliable markers to enable early estimation of clinical outcome of intensive care unit (ICU) patients are lacking. The aim of this study was to investigate, whether serum NfL levels are a possible biomarker for prediction of outcome of ICU patients.

**Methods:**

Thirty five patients were prospectively examined from admission to ICU until discharge from the hospital or death. NfL levels were measured longitudinally by a Simoa assay.

**Results:**

NfL was elevated in all ICU patients and reached its maximum at day 35 of ICU treatment. Outcome determined by modified Rankin Scale at the end of the follow-up period correlated with NfL level at admission, especially in the group of patients with impairment of the central nervous system (*n* = 25, *r* = 0.56, *p* = 0.02).

**Conclusion:**

NfL could be used as a prognostic marker for outcome of ICU patients, especially in patients with impairment of the central nervous system.

## Introduction

Neurofilaments (NfL) are structural scaffolding proteins in neurons and are known as a biomarker reflecting neuroaxonal damage in various neurological disorders [1]. NfL is composed of subunits from Nf-L [neurofilament light], Nf-M [neurofilament middle], Nf-H [neurofilament heavy], a-internexin and peripherin [2]. Through cross-bridging and interconnecting with other components of the cytoskeleton (i.e. microtubules and actin filaments), they establish a regionally specialized network that is crucial for proper nerve function [2].

Elevated levels of NfL are detectable in cerebrospinal fluid and serum and were described in diseases of the central nervous system like multiple sclerosis, dementia, stroke, traumatic brain injury etc.[1, 3, 4], but also in disorders of the peripheral nervous system like Guillain–Barré syndrome and chronic inflammatory demyelination neuropathy [5–7]. Blood levels of neurofilaments were shown to monitor and predict progression in these diseases. However, NfL levels are general indicators of neuro-axonal damage irrespective of its cause.

In patients undergoing intensive care unit (ICU) treatment, biomarkers to predict clinical outcome play an important role. Reliable prediction of outcome helps to avoid both inappropriate withdrawal of life sustaining treatment in patients with good prognosis, as well as prolonged treatment in patients without chance of adequate survival. Moreover, it helps to provide correct information for patients’ relatives, and, in case of a scarcity of resources, to allocate resources appropriately. A recent study showed that in case of resuscitation from out-of-hospital cardiac arrest more than 80% of patients admitted to an ICU have hypoxic–ischemic brain injury and about two-thirds of them die from withdrawal of life-sustaining treatment due to a predicted poor neurological outcome [8].

The aim of this study was to investigate, whether serum NfL levels are a possible biomarker for prediction of outcome of ICU patients.

## Methods

### Subjects and patients

This study was part of the prospective study on new approaches to critical illness polyneuromyopathy (Fisse et al., under review), approved by the ethics committee of the medical faculty of the Ruhr University Bochum (vote no. 16-5994). Written informed consent was obtained by patients or their legal representatives.

In this study, 35 patients of a neurologic internal medicine ICU in a university hospital of the Ruhr-University Bochum were prospectively examined clinically every 7 days from admission to ICU until discharge from the hospital or death. Inclusion criteria were ICU treatment with mechanical ventilation or catecholamine therapy for at least 24 h or sepsis or organ failure like acute respiratory distress syndrome or dialysis. Serum samples for NfL analysis were collected after admission and every 7 days during ICU treatment.

Clinical outcome was measured using modified Rankin Scale (mRS) reflecting the degree of disability ranging from 0 (no disability) to 6 (dead) [9].

### NfL measurements

Peripheral blood sampling and isolation of serum were performed according to a standardized protocol. Samples were stored at − 80 °C. NfL levels were measured at the Laboratory of University Hospital Basel by a Simoa assay. The complete protocol is described elsewhere [10]. Samples were coded randomly and were analyzed blinded for patient’s group and outcome.

### Detection of patients with critical illness polyneuromyopathy

Patients received nerve conduction studies and electromyography to detect a possible influence of critical illness polyneuromyopathy (CIPNM) on NfL levels.

CIPNM was defined as,

- Deterioration of the compound motor action potential (CMAP) amplitude during ICU stay by more than 50% and more than 1 mV compared to the baseline nerve conduction study (NCS) in at least one leg nerve, or,
- Presence of pathological spontaneous activity in the electromyography (EMG).

To show longitudinal changes in severity of NCS and EMG pathologies, the CIPNM severity score was calculated (Fisse et al., under review):

Regarding NCS of the fibular nerve:

- 1 point for demyelinating characteristics (reduced CV, prolonged DML, prolonged F-wave latency, and conduction block);
- 1 point for distal CMAP amplitude reduction below the lower limit of normal;
- 1 additional point for distal CMAP amplitude reduction > 20%;
- 1 additional point for distal CMAP amplitude reduction > 50%;
- 1 additional point for distal CMAP amplitude reduction > 70%;
- 1 additional point for lack of distal CMAP.

Regarding EMG of the tibialis anterior muscle:

- 1 point for pathological spontaneous activity in ≤ 5 of 10 tested needle layers;
- 2 points for pathological spontaneous activity in > 5 of 10 tested needle layers.

### Statistics

Statistical analyses were performed using Graph Pad Prism 8 (GraphPad Software Inc., San Diego, California, USA) and IBM SPSS Statistics 25.0.0.0 (IBM Corporation, Armonk, New York, USA). Absolute data are presented as mean ± SD or as median with range, lower and upper quartile. Differences between groups were tested by Mann–Whitney *U* test, *t* test or Chi-squared test as applicable. Probability levels (*p* values) are indicated as *, if *p* < 0.05.

## Results

### Clinical data

In 6 of the 35 patients only baseline, but not follow-up examination was possible due to death or discharge to another hospital within the first few days after admission. Mean age was 65 ± 12 years. Sepsis in 45%, resuscitation in 45%, and stroke or intracranial hemorrhage in 28% of patients were most frequent diagnoses. Central nervous system involvement (e.g. brain death after resuscitation, encephalopathy in sepsis, acute cerebral infarction) occurred in 22 (76%) of patients. Median follow-up time was 26 days (range 5–126, interquartile range 11–57). Further baseline and disease characteristics like sedation and vasopressor use are shown in Table 1.

**Table 1:** Baseline characteristics of study population (Fisse el al., under review)

	All patients (*n* = 29)
Demographics	
Age in years, mean (SD)	65 (12)
Women, *n* (%)	10 (35)
Body mass index, mean (SD)	25 (5)
Disease characteristics	
Disease duration until inclusion in days, median (IQR)	6 (8)
Days of sedation, median (IQR)	12 (15)
Days of vasopressor-use, median (IQR)	8 (13)
mRS at inclusion, median (IQR)	5 (0)
GCS at inclusion, median (IQR)	3 (2)
SOFA at inclusion, median (IQR)	9 (4)
Maximum SOFA during ICU stay, median (IQR)	10 (3)
Sepsis, *n* (%)	13 (45)
Reanimation, *n* (%)	13 (45)
Stroke or intracranial hemorrhage, *n* (%)	8 (28)
Any primary CNS disease, *n* (%)	19 (66)
Follow up time in days, median (IQR)	26 (46)
Laboratory values at inclusion, mean (SD)	
Leukocytes in 1000/µl	10.9 (4.7)
Thrombocytes in 1000/µl	250 (142)
Haemoglobin in g/dl	10.4 (2.6)
Creatine kinase in U/l	1048 (2359)
Procalcitonin in ng/ml	1.9 (5.0)
Glucose in mg/dl	156 (75)
C-reaktive protein in mg/l	122 (101)
Creatinine in mg/dl	1.1 (0.8)
Bilirubin in mg/dl	0.7 (0.8)
Oxygen partial pressure (paO2) in mmHg	109 (35)

*Mrs* modified Rankin Scale, *GCS* Glasgow coma scale, *SOFA* sepsis-related organ failure assessment score, *ICU* intensive care unit, *CNS* central nervous system

Median mRS at the end of the follow-up period up as outcome marker of all patients was 5 (range 1–6, interquartile range 2). Median duration of ICU stay was 22 days (range 8–69, interquartile range: 21). Table 2 gives further details on outcome.

**Table 2:** Outcome characteristics of study population (Fisse el al., under review)

	All patients (*n* = 29)
Death, *n* (%)	11 (38)
Outcome mRS, median (IQR)	5 (2)
Outcome GCS, median (IQR)	7 (10)
Duration of stay at ICU in days, median (IQR)	22 (21)
Duration of stay in hospital in days, median (IQR)	30 (30)
Duration of ventilation in days, median (IQR)	11 (18)
Duration from mechanical ventilation to assisted spontaneous ventilation in days, median (IQR)	6 (3)
Tracheostoma, *n* (%)	12 (41)

*mRS* modified Rankin Scale, *GCS* Glasgow coma scale, *ICU* intensive care unit

### NfL as outcome marker for ICU patients

**Fig. 1:**
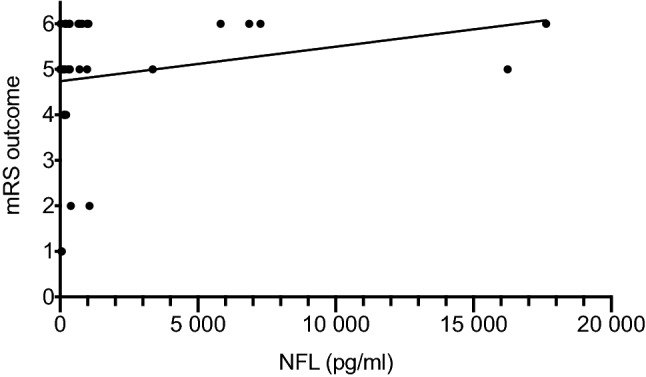
Correlation between NfL at admission and outcome mRS at end of follow-up in the whole study group (*r* = 0.58, *p* = 0.05)

**Fig. 2:**
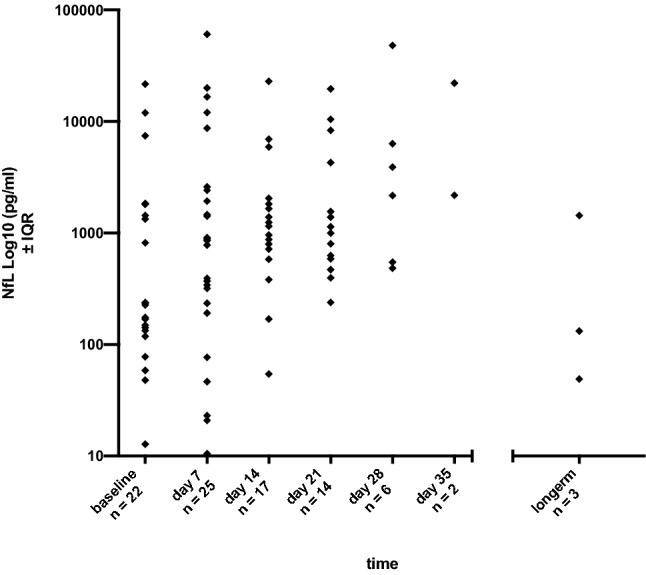
NfL course during ICU stay shown as log10 of median. Highest NfL levels were found on day 35

Outcome determined by mRS at the end of the follow -period (median 26 days) correlated with NfL level at admission in all ICU patients (*r* = 0.58, *p* = 0.05, Spearman correlation, Fig. 1). Median NfL in all ICU patients after admission was 231 pg/ml. During the ICU stay, NfL reached its maximum at day 35 with a median NfL of 12,116 pg/ml (Fig. 2; Table 3). Over time, outcome also correlated to NfL levels at day 7 (*r* = 0.49, *p* = 0.01), not on day 14, day 21 and day 28. Serum samples at day 35 were only available from 2 patients and serum samples for long term follow-up after 6 months were only available from 3 patients.

**Table 3:** NfL values (pg/ml) during ICU treatment

	Baseline	Day 7	Day 14	Day 21	Day 28	Day 35	Longterm
All patients							
Available samples	22	25	17	14	6	2	3
Median	231	857	1151	1067	3031	12,116	132
Minimum	13	11	54	238	484	2178	49
Maximum	21,689	60,591	22,909	19,608	48,101	22,054	1435
Lower quartile	130	213	650	558	531	2178	49
Upper quartile	1813	2506	1932	5290	16,759		
CNS disease							
Available samples	18	19	11	10	4	2	2
Median	528	1417	1388	1263	5106	12,116	784
Minimum	59	11	169	238	547	2178	132
Maximum	21,689	60,591	22,909	19,608	48,101	22,054	1435
Lower quartile	147	342	797	758	1385	2178	132
Upper quartile	1827	8702	5916	8864	37,654		
No CNS disease							
Available samples	4	6	6	4	2	0	1
Median	83	183	866	529	1323		49
Minimum	13	21	54	396	484		49
Maximum	175	910	1819	4275	2163		49
Lower quartile	22	23	300	414	484		49
Upper quartile	161	522	1387	3353			49

### NfL in patients with impairment of the central nervous system (CNS)

**Fig. 3:**
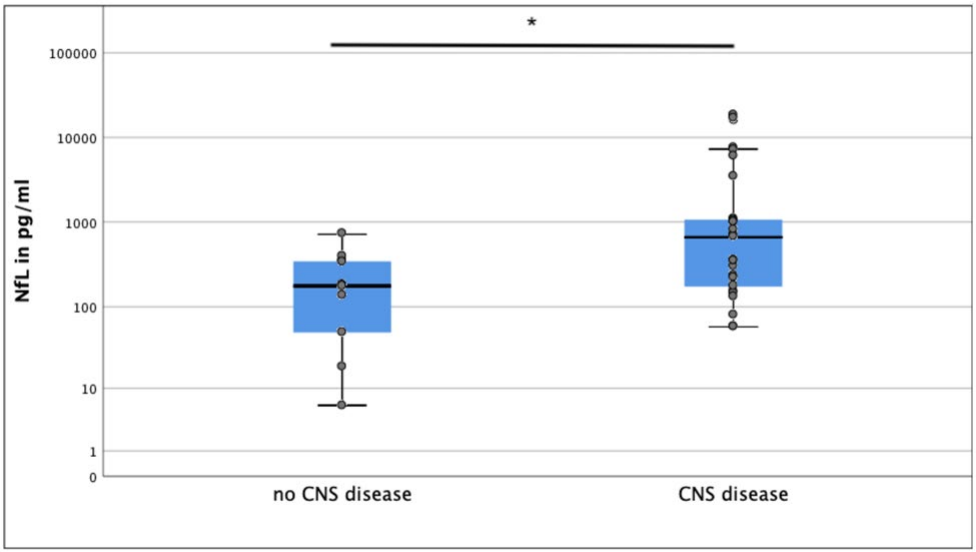
NfL levels in patients with and without CNS disease

In the group of patients with CNS damage (e.g. brain death, encephalopathy, acute cerebral infarction, *n* = 22) NfL values at admission were significantly higher compared to patients without CNS damage (*n* = 7). Median NfL at admission in patients with CNS damage was 528 pg/ml versus 83 pg/ml in patients without CNS damage (*p* = 0.02, Fig. 3, Table 3).

In this group of patients with CNS disease, there was a significant correlation between the outcome defined by mRS at the last follow-up and the NfL level at admission (*r* = 0.56, *p* = 0.02). Again, this correlation was also found on day 7 (*r* = 0.58, *p* = 0.01) and not in the following weeks.

In patients without CNS impairment, correlation between NfL at baseline and outcome NfL was not statistically significant (*r* = 0.17, *p* = 0.65). In these patients without CNS disease median NfL at admission was 83 pg/ml which above range of standard values in healthy volunteers [10, 11].

### NfL in CIPNM

69% (*n* = 20) of the 29 patients developed CIPNM, while 9 patients did not develop CIPNM. No significant differences in NfL levels between patients with CIPNM and without CIPNM were found at any timepoint. Excluding patients with CNS disease, only 6 patients with CIPNM remained. In these 6 patients with CIPNM and without CNS disease median NfL at admission was 48 pg/ml, which is in range of standard values in healthy volunteers [10, 11] and reached its maximum on day 28 with a median of 2163 pg/ml.

## Discussion

Our main finding, the correlation of NfL at baseline with the outcome mRS in ICU patients with CNS disease suggest that NfL could serve as a possible prognostic marker for these ICU patients. In some specific CNS diseases, correlation of NfL with clinical outcome was reported before, i.e. in spinal cord injury [12], traumatic brain injury [13] and multiple sclerosis [6]. The special feature of our cohort is that a broad spectrum of different neurological and non-neurological diseases was included, and NfL correlated with outcome not only in CNS-specific diseases leading to ICU treatment like for example stroke, but also in all other patients with CNS symptoms like encephalopathy and delirium irrespective of the underlying disease. If confirmed in further studies, NfL could serve as a general prognostic marker for ICU patients with CNS symptoms, like neuron-specific enolase in resuscitated patients [14]. This would be helpful in clinical context, as the extent of the neurological involvement in analgosedated patients is difficult to assess. As NfL is specific for neuronal damage, the increased NfL values in patients without clinically detectable CNS involvement compared to healthy subjects suggest that there is neglected neurological involvement also in these patients. Correlation of NfL levels with outcome shows that the degree of neurological involvement strongly influences outcome. This emphasizes the importance of interdisciplinary patient care in an intensive care unit to enable preventive or early neurological therapies if required (i.e. preservation of the circadian rhythm or physiotherapy).

Long-term data of NfL over time have shown that changes in NfL levels are associated with disability worsening in multiple sclerosis. A reduction of NfL levels at 6-month intervals was reported in multiple sclerosis patients treated with high-potency therapies [15]. To our knowledge, only scarce studies showed NfL levels in acute illness during the first weeks [16]. Our results with correlation of first NfL level with outcome and increasing NfL during the time of ICU stay suggests that NfL corresponds to the degree of axonal damage at the beginning of an acute axonal lesion, and then probably accumulates over a period of a few weeks as the correlation fades over time. The peak of NfL at day 35 probably means that for these two patients the CNS damage (one with stroke, the other one with hyperprolinemia and status epilepticus) was progressive and NfL accumulated. This could be due to an accordingly long serum half-life of several weeks which was also found in other studies about NfL kinetics [4, 17].

NfL shows axonal damage but is not specific for central nervous system. Polyneuropathies leading to increased NfL levels were described before [6, 7]; however, in our study groups, we did not find elevated NfL levels in patients with CIPNM. Critically ill ICU patients often not only have CIPNM but also CNS impairment, for example encephalopathy, which leads to high NfL levels, probably as CNS damage includes much more cells then the peripheral nerves. NfL as marker for CIPNM should therefore be examined in patients *with* CIPNM *without* CNS impairment to investigate whether NfL could be a marker for CIPNM. In our study group, however, only 6 patients with CIPNM had no CNS impairment. In these 6 patients, NfL increase during ICU treatment with maximum on day 28 could imply that NfL could still be a possible biomarker in CIPNM.

A major limitation of this study is the small number of patients. Especially NfL measurements from day 28 onwards could only be performed in only few patients, as a lot of patients had a severe disease course and died in the first weeks of ICU treatment. Further studies are, therefore, necessary to confirm our longitudinal findings. Moreover, mRS was used as outcome marker, which originally was developed as an instrument for the outcome of stroke patients. However, in our view, the mRS represents the everyday life impairment, not only through stroke, but also through other impairments.

## Conclusion

NfL could be used as a prognostic marker for outcome of ICU patients, especially in patients with CNS impairment.

## Data Availability

Data and material are available on reasonable request from the corresponding author.
